# Nondestructive Evaluation of Concrete Bridge Decks with Automated Acoustic Scanning System and Ground Penetrating Radar

**DOI:** 10.3390/s18061955

**Published:** 2018-06-16

**Authors:** Hongbin Sun, Sepehr Pashoutani, Jinying Zhu

**Affiliations:** Department of Civil Engineering, University of Nebraska-Lincoln, 1110 S 67th St., Omaha, NE 68182, USA; hbsun@unl.edu (H.S.); sepehr.pashoutani@gmail.com (S.P.)

**Keywords:** nondestructive evaluation, concrete bridge deck, acoustic scanning, GPR, delamination, deterioration

## Abstract

Delamanintions and reinforcement corrosion are two common problems in concrete bridge decks. No single nondestructive testing method (NDT) is able to provide comprehensive characterization of these defects. In this work, two NDT methods, acoustic scanning and Ground Penetrating Radar (GPR), were used to image a straight concrete bridge deck and a curved intersection ramp bridge. An acoustic scanning system has been developed for rapid delamination mapping. The system consists of metal-ball excitation sources, air-coupled sensors, and a GPS positioning system. The acoustic scanning results are presented as a two-dimensional image that is based on the energy map in the frequency range of 0.5–5 kHz. The GPR scanning results are expressed as the GPR signal attenuation map to characterize concrete deterioration and reinforcement corrosion. Signal processing algorithms for both methods are discussed. Delamination maps from the acoustic scanning are compared with deterioration maps from the GPR scanning on both bridges. The results demonstrate that combining the acoustic and GPR scanning results will provide a complementary and comprehensive evaluation of concrete bridge decks.

## 1. Introduction

Concrete bridge deck deterioration is a major concern to highway agencies. Delamination and reinforcement corrosion are two of the most common problems for reinforced concrete (RC) bridge decks. These defects will seriously affect the service life and safety of bridges. Accurate and efficient evaluation of bridge decks will help highway agencies make proper maintenance decisions and reduce repair cost. Since the Impact-Echo (IE) method was developed in the 1990’s [1,2], it has become a commonly used nondestructive testing (NDT) method to characterize concrete delaminations. However, the conventional IE test requires the contact between the sensors and concrete surface, which is time-consuming in terms of testing large concrete structures. Zhu and Popovics [3,4] developed an air-coupled IE method, which replaces the contact sensor with a microphone. Ham and Popovics [5] recently proposed using low cost micro-electro-mechanical (MEMS) microphone sensors for air-coupled sensing of concrete. Although the non-contact microphone sensors allow fast scanning speeds, the contact type impact source is still the major factor limiting test speed. Many researchers have attempted to develop automated impact sources to improve test speed, including liquid droplets [6] or ice spheres [7], a mechanically controlled hammer [8,9], and an air-coupled spark source [10]. However, these methods have various kinds of challenges in practice, such as a weak amplitude, a slow speed, or a complicated apparatus design.

In practice, a sounding method, such as a chain drag test and hammer sounding, is commonly used for the fast detection of shallow delaminations. The chain drag test does not provide quantitative and achievable data, and its interpretation relies on the operator’s judgment. To address these drawbacks of the chain drag test, the authors recently developed a multi-channel acoustic scanning system that integrates air-coupled sensing with continuous impact excitation using ball-chains [11,12]. This system can scan bridge decks at a rapid speed and generate a map of delaminations immediately after scanning. It has been validated in field testing on multiple concrete bridge decks. It should be noted that the chain drag or acoustic scanning methods are effective only on shallow delaminations.

Ground Penetrating Radar (GPR) uses electromagnetic (EM) waves to evaluate the condition of the concrete and reinforcements. EM waves can penetrate through concrete layers and will be reflected from rebars, cracks, and the bottom surface of bridge decks. GPR applications in bridge deck evaluation include identifying concrete deterioration and rebar corrosion [13], void detection [14,15], locating cracks, and delamination [16]. Various criteria for analyzing GPR data have been proposed. Most researchers used rebar layer reflection amplitude to evaluate the bridge deck condition [17,18,19], which is also used in this study. ASTM [20] suggests that an area with a more than 6–8 dB amplitude drop relative to the maximum amplitude indicates deterioration, i.e., 6–8 dB below the maximum amplitude can be used as a threshold value. However, this criterion does not apply to a bridge deck with severe deterioration or without deterioration. In addition, many factors affect rebar reflection amplitude, including rebar depth/size variation, concrete deterioration, and ambiguity in threshold determination.

Since no single NDT method can give a full picture of structure conditions, researchers explore multiple NDE techniques for comprehensive evaluation of concrete bridge decks [21,22,23,24,25]. Gucunski et al. [26] evaluated the capabilities and limitations of most commonly used NDT techniques including GPR, IE, infrared thermography, chain dragging, and ultrasonic surface waves. Each NDE technique was evaluated from five perspectives: accuracy, speed, repeatability, ease of use, and cost. This study presents an application of two complementary NDT techniques: the acoustic scanning method and the GPR method for a condition assessment of bridge decks. These two methods are complementary in physics principles and depth of detection, and both methods provide relatively fast test speeds compared to other contact type NDT methods. The acoustic scanning method provides rapid mapping of shallow delaminations (up to 5–6 cm). GPR antennas used for concrete structure or bridge scans have penetration depths of 0.3–0.5 m. For concrete bridge deck assessment, GPR reflection signals from bridge surfaces to top layer rebar meshes are typically used to evaluate concrete deterioration and rebar corrosion. Signal processing algorithms are discussed for both acoustic and GPR signals. Both methods were deployed on concrete decks of a straight bridge and a curved highway intersection bridge.

## 2. Acoustic Scanning Method

### 2.1. Acoustic Scanning System

The acoustic scanning system includes an array of excitation sources and acoustic sensors (microphones), a data acquisition device, and a GPS positioning device. A newly developed ball-chain was used as the excitation source [11]. The ball-chain is made of 12.7 mm and 15.9 mm diameter brass balls with a spacing of 25 mm. Smaller balls (12.7 mm) are used for delaminations with higher frequency, and larger balls (15.9 mm) are for delaminations with lower frequency. In a previous study [11], the conventional steel link-chain and the ball-chain were tested on solid concrete surfaces and various delaminations. Comparison of these testing results shows that the ball-chain gave signals with higher signal-to-noise (S/N) ratios than the traditional steel link-chain. The difference is caused by different impact mechanisms from the ball-chain and link-chain. When a ball-chain is dragged on concrete surface, the balls jump and impact the surface randomly, and the impacts excite stress waves in concrete. The received acoustic signals are similar to the signals in the impact-echo test. In the conventional chain drag test, the link-chain mainly slides on the concrete surface, where the surface friction is the driving force for acoustic signals.

**Figure 1:**
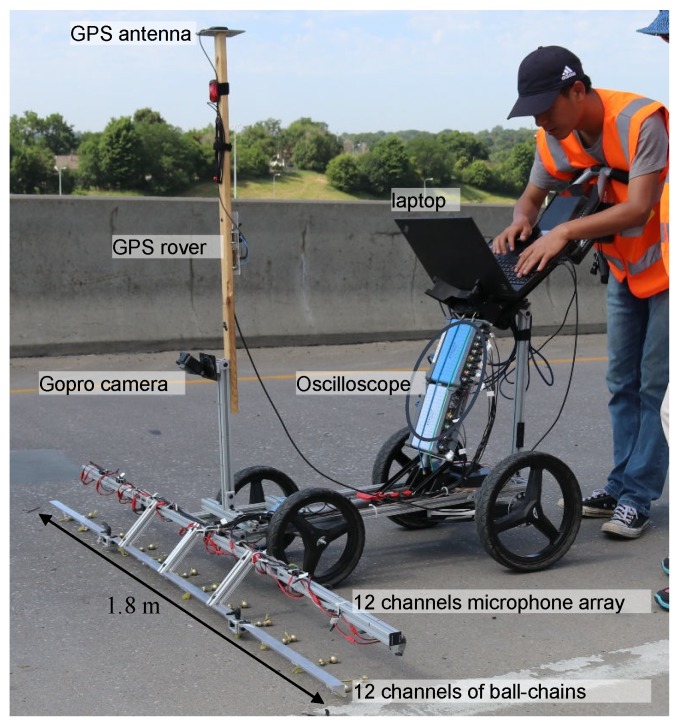
12-channel acoustic scanning cart.

Low-cost MEMs (micro-electro-mechanical systems) microphones (Adafruit SPW2430) with a nominal frequency range of 100 Hz to 10 kHz (actual frequency range is broader) were used as the acoustic sensors. The microphones and the ball-chains were installed on a 1.8 m long scanning frame (see Figure 1). The testing frame includes 12 sensing channels with a spacing of 0.15 m, and each channel includes one MEMs microphone and one ball-chain. The acoustic signals received by the microphone array were digitalized by two oscilloscopes (PICO4824 and PICO5444) with a sampling rate of 100 kHz and transferred to a computer. Meanwhile, a Real Time Kinematic (RTK) GPS system (Piksi GPS, Swift Navigation, Inc., San Francisco, CA, USA) provided real-time positioning during the scanning. A LabVIEW program was designed to control the data acquisition of acoustic signals, and positioning data. When the data collection was finished, a two-dimensional scanning image was generated using the following signal processing algorithm to map the delaminations. The scanning cart will scan bridge decks in a normal walking speed (about 1.2 m/s). With a scanning frame width of 1.8 m, the system can scan an area of 2.16 m${}^{2}/$s. The longitudinal spatial resolution depends on the scanning speed. Based on our work, at walking speed, this system will give a spatial resolution of 2–5 cm. The testing speed effect is discussed in detail in a previous study by the authors [11].

### 2.2. Acoustic Signal Processing Algorithm

**Figure 2:**
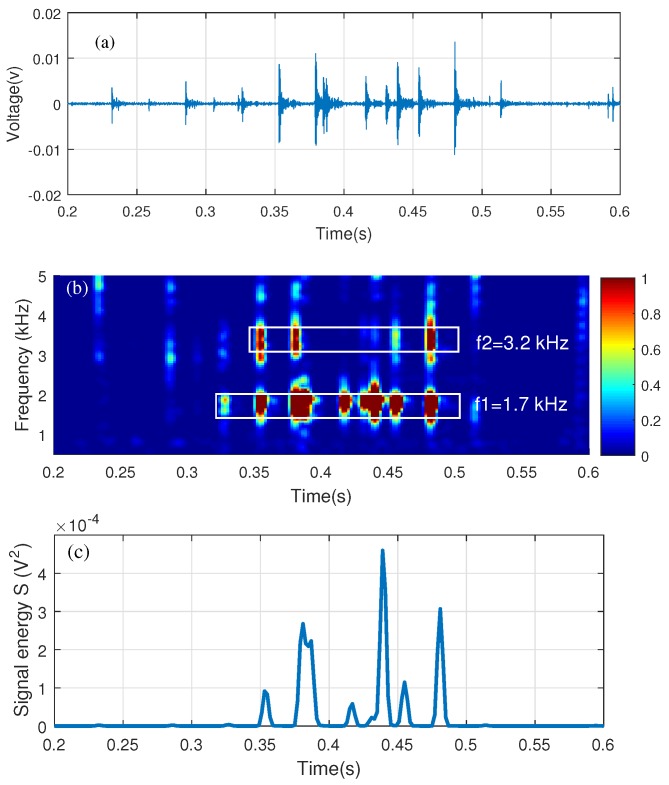
Ball-chain test results: (**a**) time-domain signal; (**b**) STFT spectrogram with normalized amplitude; (**c**) signal energy.

Acoustic signals were recorded continuously in a streaming mode during scanning. Delamination responses are usually analyzed in the frequency domain. In order to show acoustic frequency content change over time, the time-domain signals were processed by Short Time Fourier Transform (STFT). The STFT algorithm will convert the continuous time domain signal into a 2-D dataset (STFT spectrograms) with the x-axis as the signal time and the y-axis as the frequency. Figure 2a shows the time-domain signal of one scan for a ball-chain dragging on a delamination. Each spike in the time domain represents one impact on the concrete surface, and these spikes correspond to the vertical bright strips in the STFT spectrogram (Figure 2b). If a delamination presents, the resonance responses of this delamination will appear on these strips. According to a semi-analytical analysis of resonance frequencies for square concrete delaminations by Kee and Gucunski [27], the resonance frequency ranges from 0.5 to 5 kHz for delaminations with a depth of 2.5–7.5 cm and width of 0.2–1 m. Therefore, in this paper, the resonance responses in 0.5–5 kHz frequency range are used to identify the existence of delaminations.

Because each time domain signal represents a 1-D linear scan on bridge, a signal energy parameter *S* is defined to compress the 2-D STFT spectrogram to 1-D format. The signal energy *S* is calculated by integrating the power spectrum over the frequency range [$f_{1}$, $f_{2}$], which takes the summation of the square of the spectrogram in Figure 2b along the frequency axis, and can be represented by [12]:

(1) $$S\left(t\right)=\int_{f_{1}}^{f_{2}}{\left|X(f,t)\right|}^{2}df$$

where X(f,t) is the 2-D dataset for the spectrogram in Figure 2b and the frequency integration range is 0.5–5 kHz. The signal energy curve is shown in Figure 2c. Here, the microphone output signal has a unit of Volt (V) and *S* has a unit of $V^{2}$. In the figure, the signal energy has high amplitudes when delamination responses exist. The process was repeated for each scanning channel and a signal energy curve S(t,i) was exacted for the *i*th channel. The signal energy curves from all channels were then combined to form a 2-D dataset S(t,n) (*n* is the total channel number). The time axis and the channel number axis would be replaced by the position coordinates to generate a 2-D delamination map. Since the signal energy is a very small value, it is convenient to express it in decibels (dB) using the following equation:

(2) S(dB)=10log(S).

### 2.3. GPS Positioning

The acoustic scanning system uses an RTK GPS system (Piksi GPS, Swift navigation, Inc.) for real time positioning. The positioning system has two identical units with one unit as the base and the other one as the rover installed on the scanning cart. A coordinate system is built using the GPS base as the origin, the east direction as the *X*-axis, the north direction as the Y-axis, and the upward direction along the ellipsoid normal as the *Z*-axis. The relative position between the GPS rover and the base is defined as the scanning cart coordinate (*x*, *y*, *z*). The coordinates of the GPS rover were recorded continuously at a rate of ten times per second (10 Hz). However, it is convenient to show the delamination map using a local coordinate system instead of the GPS global coordinate system. In this local coordinate system, the longitudinal direction of a bridge deck is denoted as the *X${}^{\prime }$*-axis and the transverse direction of the deck as the *Y${}^{\prime }$*-axis. The *Z${}^{\prime }$*-axis is the same with the global coordinate system.

**Figure 3:**
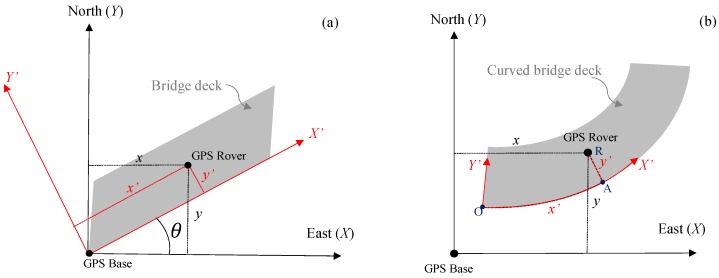
Positioning coordinates transformation on (**a**) the straight bridge and(**b**) the curved bridge.

For a straight bridge deck, the local coordinates (*x${}^{\prime }$*, *y${}^{\prime }$*, *z${}^{\prime }$*) of the scanning cart were transferred from the global coordinates (*x*, *y*, *z*) using the rotation of axes (see Figure 3a). The transformation is shown in Equation (3).

(3) $$\left[\begin{array}{c} x^{\prime }\\ y^{\prime }\\ z^{\prime } \end{array}\right]=\left[\begin{array}{ccc} \cos\theta & \sin\theta & 0\\ -\sin\theta & \cos\theta & 0\\ 0 & 0 & 1 \end{array}\right]\left\{\begin{array}{c} x\\ y\\ z \end{array}\right\}$$

where θ is the angle between the deck longitudinal orientation and the east direction.

For a curved bridge, test results will be presented in a 2D, uncurled format with distance and width as axes. Therefore, the local coordinate system was built using the curved deck edge as the *X${}^{\prime }$*-axis and the direction normal to the curved edge as the *Y${}^{\prime }$*-axis (see Figure 3b). First, the curved deck edges (*X${}^{\prime }$*-axis) were surveyed with the RTK GPS. Then the perpendicular distance from the GPS Rover to the curved deck edge (RA) was denoted as $y^{\prime }$ and the length of the curve AO as $x^{\prime }$.

In the 2-D dataset S(t,i), the time axis *t* was converted to the coordinates $x_{t}^{\prime }$ of the scanning path in $X^{\prime }$ axis, and the channel axis *i* was converted to the coordinates $y_{i}^{\prime }$ in $Y^{\prime }$ axis. The values of $x_{t}^{\prime }$ and $y_{i}^{\prime }$ were calculated based on the local coordinates $\left(x^{\prime },y^{\prime }\right)$ as follows:

(4) $$\begin{array}{ccc} x_{t}^{\prime } & = & interp(t_{G},x^{\prime },t_{A}) \end{array}$$

(5) $$\begin{array}{ccc} y_{i}^{\prime } & = & y^{\prime }+y_{i,o}\;i=1,2,\cdots ,n \end{array}$$

In Equation (4), interp represents the linear interpolation process of using the GPS time stamp $t_{G}$ and positioning coordinates $x^{\prime }$ to obtain the location for $t_{A}$ of the acoustic signal. In Equation (5), $y_{i,o}$ are the offset distances of the 12 acoustic sensors to the GPS rover, which was installed at the middle position of the scanning frame (see Figure 1). The offset distances are from −0.9 to 0.9 m with 0.15 m spacing. The altitude deviation of the deck (in the Z direction) was neglected. For a 90 m long bridge deck with 0.5 m altitude change, the maximum positioning error in the longitudinal direction was only 0.01 m if the altitude deviation is neglected.

## 3. GPR Scanning System and Signal Processing

**Figure 4:**
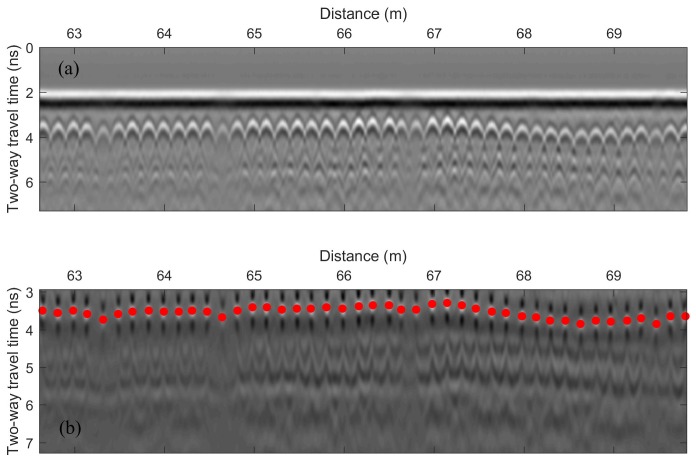
GPR B-scan images from a bridge deck. (**a**) Raw data. (**b**) Migrated signals with identified rebar locations.

**Figure 5:**
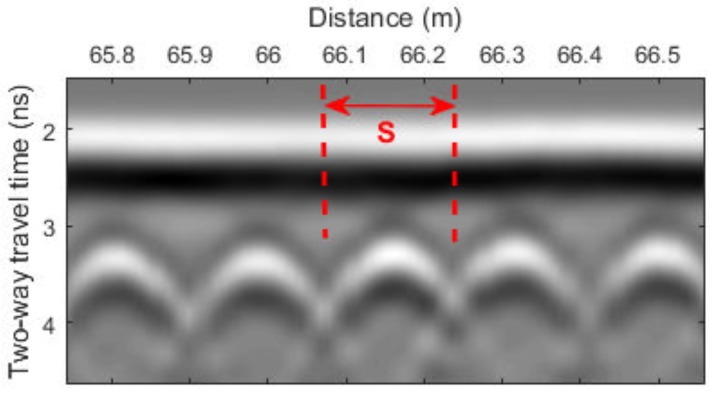
Averaged direct coupling amplitude along the distance “S” (half of rebars spacing from each side of the rebar) was used to normalize the rebar amplitude.

**Figure 6:**
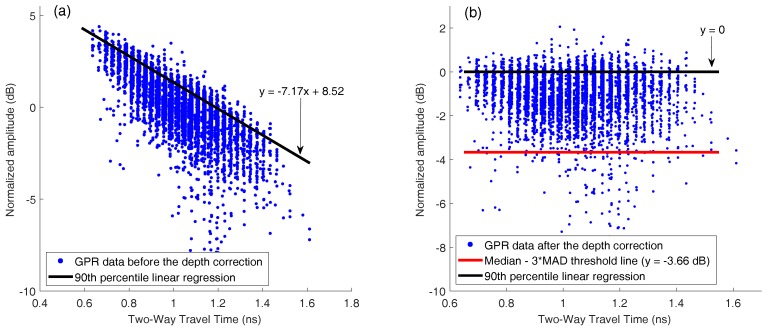
Scatter plot of GPR data points (**a**) before and (**b**) after amplitude-depth correction and 90th percentile linear regression from the bridge with sound concrete.

**Figure 7:**
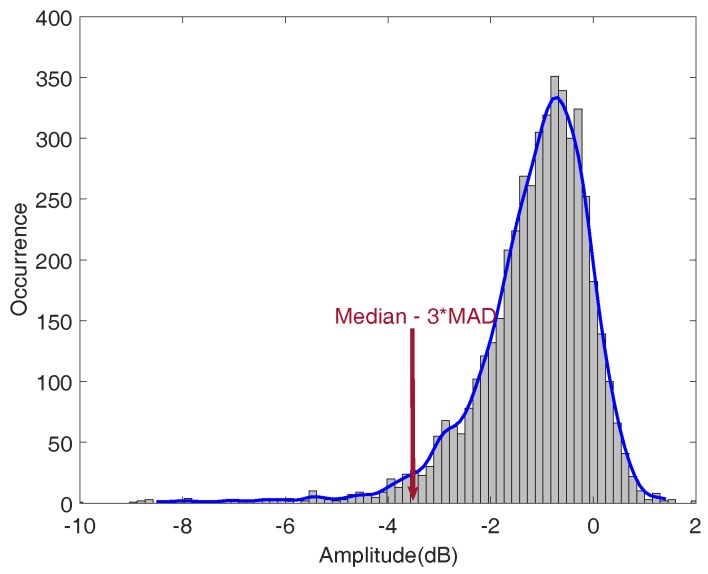
Histogram of the GPR data of sound bridge.

A GSSI SIR-3000 system with a 1.5 GHz ground coupled antenna was used for the bridge deck evaluation. In all tests, the vertical two-way travel time was set to 15 ns, which gives enough time to receive echo signals from the bottom surface of bridge decks. The longitudinal data resolution of the system was fixed at 0.011 m. An RTK GPS and a wheel encoder were used for positioning. GPR scanning was implemented along the longitudinal direction of bridge decks. The collected data were then transferred to a computer for further signal processing. A MATLAB program was developed for GPR signal processing. The processing steps are as follows:

1. Migration (auto-focusing): Due to beam spreading effect, reinforcements appear as hyperbolas in the GPR B-scan (see Figure 4a). It was observed that the peak amplitude of rebar reflection does not always occur at the hyperbola vertex. The strongest reflection may occur on the tails of hyperbolas because of variation in direct coupling amplitude or unknown anomalies that surrounded rebars. Therefore, migration algorithm is used to focus the rebar reflection to the vertex point. Migration is a widely used mathematical method in geophysics to identify locations of buried objects underground. Several migration algorithms have been discussed and evaluated by Özdemir, et al. [28]. In this work, Stolt migration (the frequency-wavenumber method) algorithm was used for auto-focusing the rebar reflection due to its fast computation and good signal-to-clutter ratio [29].
2. Automated rebar picking: Although many GPR post-processing softwares have the function to detect rebar locations and extract the GPR amplitudes automatically, the authors found that the accuracy is not satisfying in long bridges with many rebars. In this study, we developed a MATLAB program for automatic rebar detection. On migrated images, rebar reflections are identified by choosing the local maximums. (see Figure 4b).
3. Data normalization: GPR signals are commonly normalized to a reference amplitude. Typically, the reference amplitude uses 32,767 ($2^{15}-1$) for 16 bit data and 2,147,483,647 ($2^{31}-1$) for 32 bit data and then the GPR data is converted to decibel scale [30]. This method does not consider the variation of surface reflections. Since an EM wave first encounters the bridge surface, any surface defects and anomalies may affect the rebar reflection (the hyperbola amplitude). Therefore, the average surface direct-coupling amplitude on top of each hyperbola (see Figure 5) was used to normalize the corresponding rebar reflection amplitude after the migration.
4. Amplitude correction due to depth variation: Both rebar depth and concrete deterioration affect the GPR amplitude and travel time. Variation in rebar depth is not related to the concrete deterioration or rebar corrosion condition. To correct the depth variation effect, the relationship between rebar depth and GPR amplitude can be obtained. A previous study has shown that the rebar reflection amplitude in decibel scale has a linear relationship with the rebar depth [31]. However, the actual rebar depth is not usually available in practice. Dinh et al. [31] used GPR datasets collected from sound bridge decks to establish a relationship between amplitude and two-way travel time (TWTT) of GPR signals. This relationship was then used to correct the depth effect for GPR signals from other bridges. In this study, GPR data collected on a sound concrete bridge deck were analyzed using Steps 1–3. Figure 6a shows the GPR signal amplitude versus the two-way travel time on the sound bridge. According to [32], the 90th percentile linear regression is used to build the amplitude–TWTT correction relationship that corresponds to the top 10 percent of rebar amplitudes for all TWTTs. In Figure 6, the 90th percentile line for a sound bridge deck has a slope of −7.17 dB/ns. For a sound bridge deck, this slope represents the effects of rebar depth variation; while in a bridge deck with deterioration, it also includes additional attenuation of EM waves caused by concrete deterioration. Therefore, the concrete deterioration can be evaluated by applying depth correction to all data points through subtraction of this relationship. The amplitude after this correction represents the effects of the rebar size, concrete deterioration, and corrosion condition. After such correction, GPR amplitude vs. TWTT from a sound bridge deck should have a slope near zero. A negative slope or lower amplitude after correction may indicate deterioration of concrete and/or corrosion of rebars.
5. Threshold: In order to determine a threshold value for generating the GPR attenuation map, a histogram of the GPR data from the sound bridge deck is shown in Figure 7. For a bridge in excellent condition, the rebar amplitudes after TWTT correction should follow a normal distribution. However, minor defects of concrete or rebar corrosion will slightly skew the amplitude distribution to the negative side, and there are outliers in the dataset. In order to detect outliers in a dataset, Leys et al. [33] recommends using Median Absolute Deviation (MAD) around the median. The MATLAB function *isoutlier* is used to detect outliers that are away from the median by 3-fold-scaled MAD. Using this function, a value of −3.66 dB was chosen as the threshold value that indicates concrete starting to deteriorate.

## 4. Results

### 4.1. A Straight Concrete Bridge Deck

**Figure 8:**
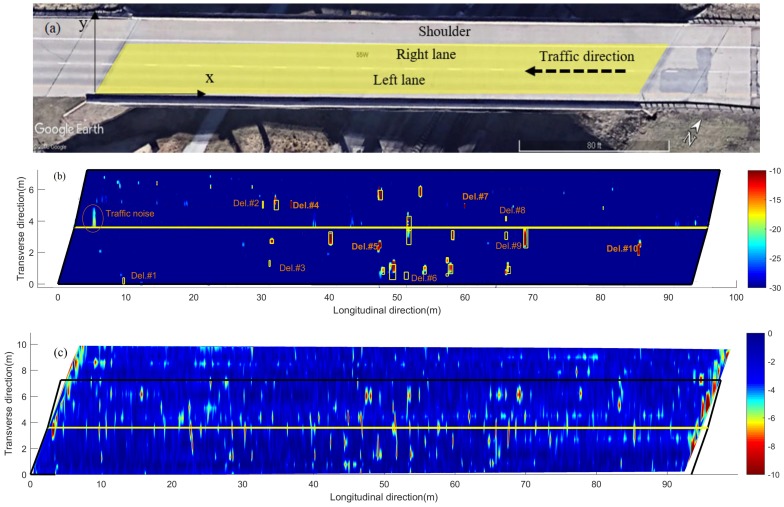
Straight concrete bridge deck: (**a**) Google Earth image; (**b**) acoustic scanning image; (**c**) GPR attenuation image.

A 90 m long concrete bridge deck in Lincoln Nebraska was tested (the yellow area in Figure 8a) using the automated acoustic scanning system and the GPR system.The right lane was scanned using the acoustic scanning system in November 2016 and the left lane was scanned in July 2017. The GPR system scanned both lanes in July 2017, with a line spacing of 0.6 m. Manual chain drag test was also conducted during the acoustic scanning, and the results were used as a validation of the acoustic scanning. A coordinate system was built in the figure with the *X*-axis representing the longitudinal direction of the bridge and the *Y*-axis representing the transverse direction. The GPS base unit was placed at the origin (0,0), and the rover unit was installed on the scanning cart. The (*x*, *y*) coordinates of the rover were measured using the relative position between the rover and the base.

Figure 8b shows the acoustic scanning results of the concrete bridge deck, and the lane divider is shown as the yellow line in the figure. Red spots are the delaminations in the bridge deck, and the blue areas represent sound areas. These solid areas have an average amplitude of −60 dB. In order to highlight the delaminations with good contrast, a color scale of −30 to −10 dB is used in the figure. The positions of these delaminations match well with the manual chain drag results, which are shown in yellow rectangles. However, the acoustic scanning results missed several delaminations (Del. #1, #2, #3, #6, #8, #9), which were identified by the manual chain drag. The scanning detected four extra delaminations (Del. #4, #5, #7, #10), which were missed by the manual chain drag, and multiple scans along the same paths confirmed these four delaminations. The left lane (passing lane) shows more delaminations than the right lane. The locations and sizes of all delaminations are identified by a boundary tracing algorithm [34] based on the MATLAB function *bwboundaries* [35]. The total delamination area is about 5.50 m${}^{2}$. Compared with the total scanned area 670 m${}^{2}$, the delamination area percentage is about 0.82%. When the right lane was tested, the left lane was open to traffic. The traffic noise is shown in the right lane near the lane divider. The bridge was closed when the left lane was tested. Therefore, there is no traffic noise shown in the left lane image.

**Figure 9:**
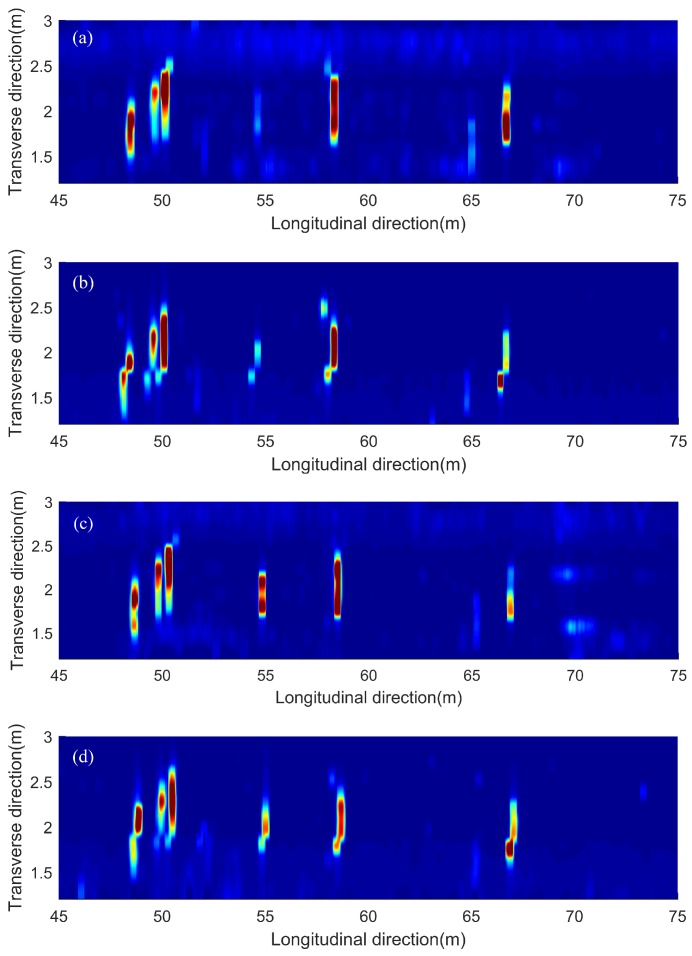
Scanning results of a 30 m long bridge deck: (**a**) Scan #1; (**b**) Scan #2; (**c**) Scan #3; (**d**) Scan #4.

To investigate the repeatability of the acoustic scanning system, four scans were collected on the same area of the bridge deck in Figure 8a. Scans #1 and #3 were tested along the same direction and Scans #2 and #4 were from the opposite direction. In Figure 9, a scanned area of 30 m long by 1.75 m wide with several delaminations was chosen to compare the four scanning results. By comparing the results of the four scans, all four images agree well with each other in terms of the locations, shapes, and dimensions of all major delaminations. There are some minor differences for small size delaminations, such as the delamination near 55 m position, among the four results. Scans #3 and #4 clearly detected this delamination with high amplitudes, while Scans #1 and #2 show this delamination with lower amplitude. Another delamination around 67 m is shown in Scans #1 and #4 more clearly than in Scan #3. Therefore, the acoustic scanning system provides satisfactory repeatability. If possible, multiple scans are recommended to provide more accurate evaluation of bridge deck conditions, especially for small delaminations.

**Figure 10:**
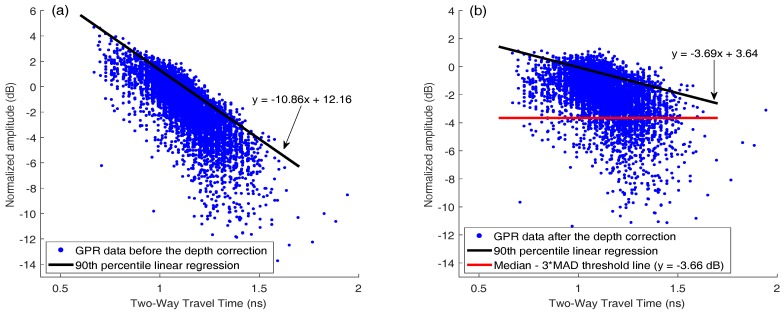
Scatter plot of GPR data points from the straight bridge (**a**) before and (**b**) after amplitude-depth correction, and threshold line.

Figure 10 shows GPR signal amplitude vs. TWTT data for the bridge, before and after applying depth correction. In this bridge 12.1% of the data points are below the threshold value −3.66 dB (red line in Figure 10b). Because rebar layouts are usually uniform in bridge decks, this number 12.1% also represents the percentage of deck area in deterioration condition. Figure 8c shows the GPR scanning images of the bridge including two lanes and the shoulder. The color scale from −10 to 0 dB was used to show normalized rebar reflection amplitudes after depth correction. Red spots represent low GPR amplitudes or high attenuation. Attenuation in rebar reflection amplitude can be due to concrete deterioration, rebar corrosion, high moisture content, cracks, or a combination of all factors. In this bridge, it is clear that along the joints, GPR signals show high attenuation. It is probably caused by high chloride content and moisture induced by accumulation of deicing salt. In addition, there are many isolated high attenuation areas (red spots) spreading over the entire bridge deck.

**Figure 11:**
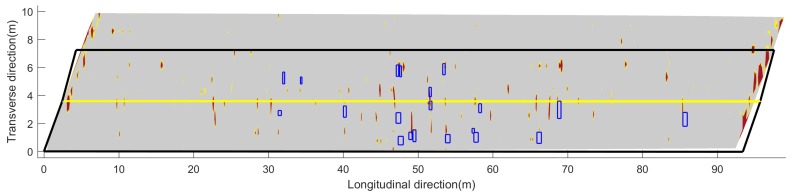
Combined results of acoustic scanning and GPR scanning.

In order to compare the acoustic scanning results and the GPR scanning results, Figure 11 shows the combined image of GPR results and the acoustic scanning results. The GPR scanning image is the same as in Figure 8c but uses a different color map. The gray area represents a good concrete deck and the red spots indicate concrete deterioration or rebar corrosion. The delaminations detected by the acoustic scanning are plotted by the blue rectangles. Both GPR results and acoustic scanning results show many defects in the region of 40–60 m. The positions of defects detected by two scanning methods are not completely matched. It is noted that the GPR detected more defects than the the acoustic scanning, such as defects in the region of 10–30 m, 65–75 m, and in the right (upper) lane. The acoustic images (Figure 8b and Figure 11) also show more delaminations in the left lane than in the right lane. The difference could be because acoustic scans on the right lane was performed eight months before other scans. Although temperature and humidity do not affect acoustic scan results at this low frequency range (<5 kHz), the bridge deck may experience more deterioration after eight months with traffic. The GPR results indicate that the two joints are deteriorated, which were not detected by the acoustic method. The overall deterioration area percentage identified by acoustic and GPR tests are 0.82% and 12.1%, respectively. The discrepancy is mainly due to different sensing mechanisms between the acoustic and the GPR tests. The acoustic sensing method can only detect near surface delaminations. For corrosion-induced delaminations, they usually represent the late stage of concrete deterioration. While in GPR tests, the EM wave can penetrate concrete. Both rebar corrosion and concrete deterioration will contribute to GPR signal attenuation. Therefore, GPR attenuation is more sensitive to early stage deterioration than the acoustic test.

### 4.2. Curved Intersection Concrete Bridge Deck

**Figure 12:**
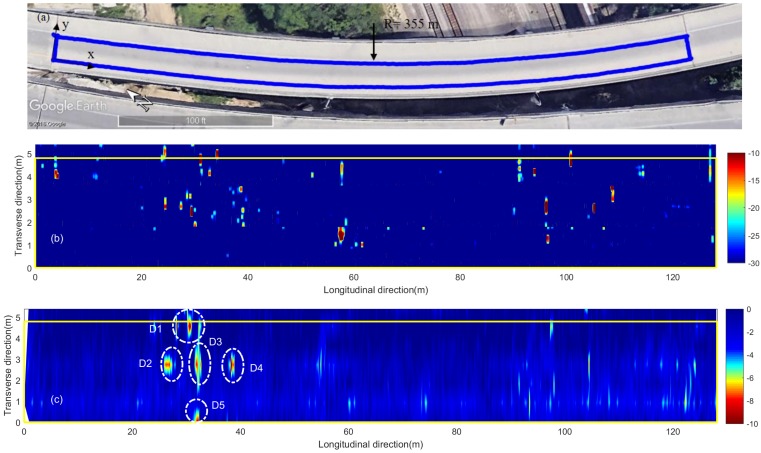
Curved concrete bridge deck: (**a**) Google Earth image; (**b**) acoustic scanning image; (**c**) GPR attenuation image.

The second tested bridge is a curved highway intersection ramp bridge in Omaha, Nebraska. This bridge is about 240 m long with a curvature radius of about 355 m. A 126 m long driving lane segment (blue curved rectangle in Figure 12a) was tested using the acoustic scanning system and GPR in July 2017. The acoustic scans have a lateral resolution of 0.15 m. Because of time constraints of the field testing, the GPR scans used a large lateral spacing of 0.9 m.

Figure 12b shows the acoustic scanning results of the curved bridge deck in a straight manner. The yellow lines represent the lane boundaries. The image color scale (−30 to −10 dB) is the same as that used in Figure 8b. In Figure 12b, most delaminations are located near the lane center and lane inner boundary (upper yellow line in the figure). Visual inspections confirmed that the delamination positions (lane center and inner boundary) match the two wheel paths on this deck. The total delamination area is 19.7 m${}^{2}$, which corresponds to 2.8% of the total scanning area 691.7 m${}^{2}$.

**Figure 13:**
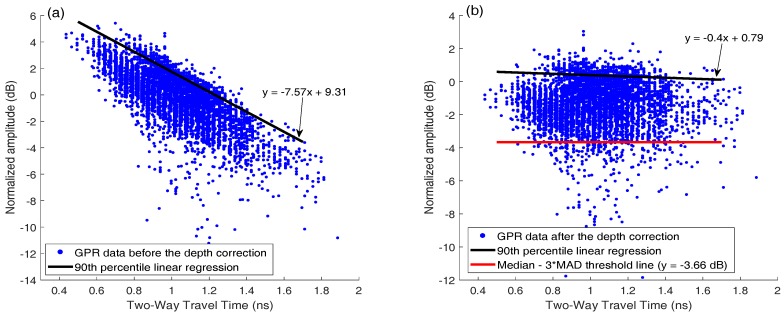
Scatter plot of GPR data points from the curved bridge (**a**) before and (**b**) after amplitude- depth correction, and threshold line.

Figure 13a shows the GPR amplitudes of rebar reflection and TWTT from the curved bridge. After depth correction, 4.5% of data points in this bridge deck are below the threshold (−3.66 dB). Figure 12c shows the GPR scanning image. The same color scale (−10 dB to 0 dB) as in Figure 8c is used. Five major deterioration areas (D1–D5) were detected on the bridge deck using the threshold amplitude. No deterioration was detected along the joints in this bridge deck.

Comparing to acoustic scanning image in Figure 12b, the deterioration areas (D1, D2, D3, and D4) on the GPR image roughly match the cluster of scattered delaminations located between 20 and 40 m. However, the acoustic test detected a major delamination around 58 m, and some small delamintions around 95–110 m. They are not shown in the GPR image. A possible explanation is that the GPR scan used a line spacing of 0.9 m, while the acoustic system has a smaller lateral spacing of 0.15 m. The large line spacing of GPR scans gives a poor lateral resolution and may miss defects between scan lines. The GPR image shows deterioration areas D5 and some minor deteriorations around 90–125 m, which are not shown in the acoustic scanning image. Recorded video images did not show any surface defects either. GPR B-scan images in these regions show weak reflections from rebars, which may indicate rebar corrosion at an early stage that did not cause severe concrete delamination yet.

## 5. Discussion

The area percentage of deterioration detected by the acoustic system and GPR are 0.82% and 12.1% in the straight bridge, and 2.8% and 4.5% in the curved bridge, respectively. In both cases, GPR, compared with the acoustic system, detected a larger area of deterioration. As explained in the previous section, GPR can detect rebar corrosion and concrete deterioration at early stages, while the acoustic system only detects shallow delaminations caused by either surface cracks or a late stage of rebar corrosion. On the other hand, the difference between acoustic and GPR results is larger in the straight bridge than in the curved bridge. Based on visual inspection, the straight bridge deck has many transverse cracks, and most delaminations occur in small areas around these cracks. Therefore, the detected delaminations are shown as narrow vertical strips in Figure 8b. Although the delaminations are widely scattered on the bridge deck, the total delamination area is low, which may underestimate the deck area needing repair. In the curved bridge, there were many surface voids and potholes, which were shown as defects in the acoustic scanning, and increased the defect area. From GPR scanning images, the curved bridge did not show clear joint deterioration, and GPR amplitudes also show less scattering (Figure 13b) than in the straight bridge. Therefore, based on GPR scanning results, the deck condition is better in the curved bridge than in the straight bridge.

Both acoustic and GPR systems have high spatial resolution in the scanning direction. At normal walking speed, the longitudinal resolutions are about 2–5 cm for acoustic scanning and 0.5–2 cm for GPR scanning, respectively. Since multi-channel microphones are used in the acoustic system, a lateral spacing of 15 cm or smaller can be reached. A multi-channel GPR system is not widely used in practice due to its high cost. With the common single channel GPR system, many line scans are needed to get a full coverage of a bridge deck, with a typical line spacing of 0.5–0.6 m. Although the GPR test is more sensitive to defects in early stages than the acoustic test, the acoustic scanning system with high spatial resolution and high sensitivity to surface defect provides complementary information for bridge deck evaluation.

## 6. Conclusions

In this study, two NDT methods were studied to evaluate concrete bridge decks. A recently developed acoustic scanning system was applied in field testing for rapid delamination mapping. The GPR scanning was used to characterize concrete deterioration and rebar corrosions in concrete bridge decks. Based on the acoustic and GPR scanning results, the following conclusions can be drawn:

1. The acoustic scanning enables rapid and high-resolution delaminations mapping on concrete bridge decks with a test speed of about 1.5 m/s (scan area of 2.7 m${}^{2}$ per second). The delaminations detected by the acoustic scanning matched well with the manual chain drag results. The acoustic scanning also detected extra delaminations, which were missed by the manual chain drag.
2. In order to eliminate the effect of surface anomaly reflection, GPR signals were normalized by the surface reflection amplitudes.
3. A relationship between GPR rebar reflection amplitude and two-way travel time was established based on data collected from a concrete bridge deck of good quality. A 90th percentile linear regression was derived, and this relationship was used to correct the effect of rebar depth variation on GPR reflection amplitude. In future, more GPR data from sound concrete bridge decks will be used to validate the obtained results.
4. A threshold value of GPR amplitude was determined based on a dataset collected from a good quality bridge deck using statistical analysis. GPR attenuation maps were then generated using a colormap based on the threshold value. More data are needed to investigate if the threshold value may apply to other types of bridge decks, including asphalt overlaid decks.
5. The acoustic scanning has a spatial resolution about 2–5 cm in the longitudinal direction, depending on the testing speed. The lateral resolution is 15 cm, with a coverage width between 1.2 and 3.6 m (lane width), depending on the number of channels. Traffic noise has a slight effect on the scanning results (see traffic noise in Figure 8b). Based on tests in a former study [12], the traffic noise amplitude is about 10% of the delamination response. Therefore, it is usually differentiable from the delamination responses. Sound insulation can be developed in future work to minimize the traffic noise effect and inter-channel interference.
6. Comparison between the acoustic scanning and GPR results shows that these two methods provide complementary information about conditions of bridge decks. Although both methods may detect some common defects, the acoustic scanning mainly identify shallow delaminations, while the GPR is able to evaluate concrete deterioration and rebar corrosion at early stages. Therefore, GPR in general detects more flaws than the manual chain drag and acoustic scanning methods. GPR also detected deterioration along bridge joints (see Figure 8c), which could not be detected by acoustic scanning. Since both sensing methods are non-contact and allow relatively fast scanning, combination of the acoustic scanning and GPR scanning will provide more efficient and comprehensive in-situ evaluation of reinforced concrete bridge decks than a single NDT method can offer.
